# Rest perfusion abnormalities in hypertrophic cardiomyopathy: correlation with myocardial fibrosis and risk factors for sudden cardiac death

**DOI:** 10.1016/j.crad.2014.12.018

**Published:** 2015-05

**Authors:** A. Chiribiri, S. Leuzzi, M.R. Conte, S. Bongioanni, K. Bratis, L. Olivotti, C. De Rosa, E. Lardone, P. Di Donna, A.D.M. Villa, F. Cesarani, E. Nagel, F. Gaita, R. Bonamini

**Affiliations:** a King's College London, Wellcome Trust/EPSRC Medical Engineering Centre, Division of Imaging Sciences, St Thomas' Hospital, UK; b Department of Internal Medicine, University of Torino, Italy; c Division of Cardiology, Cardinal Massaia Hospital, University of Torino, Asti, Italy; d Division of Cardiology, A.O. Ordine Mauriziano di Torino Presidio Umberto I, Torino, Italy; e Department of Cardiology, Santa Corona Hospital, Pietra Ligure, Italy; f Department of Radiology, Cardinal Massaia Hospital, Asti, Italy

## Abstract

**Aim:**

To measure the prevalence of abnormal rest perfusion in a population of consecutive patients with known hypertrophic cardiomyopathy (HCM) referred for cardiovascular MRI (CMR), and to assess any associations between abnormal rest perfusion and the presence, pattern, and severity of myocardial scar and the presence of risk factors for sudden death.

**Materials and methods:**

Eighty consecutive patients with known HCM referred for CMR underwent functional imaging, rest first-pass perfusion, and late gadolinium enhancement (LGE).

**Results:**

Thirty percent of the patients had abnormal rest perfusion, all of them corresponding to areas of mid-myocardial LGE and to a higher degree of segmental hypertrophy. Rest perfusion abnormalities correlated with more extensive and confluent LGE. The subgroup of patients with myocardial fibrosis and rest perfusion abnormalities (fibrosis+/perfusion+) had more than twice the incidence of episodes of non-sustained ventricular tachycardia on Holter monitoring in comparison to patients with myocardial fibrosis and normal rest perfusion (fibrosis+/perfusion–) and patients with no fibrosis and normal rest perfusion (fibrosis–/perfusion–).

**Conclusions:**

First-pass perfusion CMR identifies abnormal rest perfusion in a significant proportion of patients with HCM. These abnormalities are associated with the presence and distribution of myocardial scar and the degree of hypertrophy. Rest perfusion abnormalities identify patients with increased incidence of episodes of non-sustained ventricular tachycardia on Holter monitoring, independently from the presence of myocardial fibrosis.

## Highlights

• 30% of patients with HCM have perfusion abnormalities related to scar.
• No rest perfusion abnormalities were observed in areas of viable myocardium.
• Scar-related perfusion abnormalities were associated with the incidence of NSVT.

## Introduction

Sudden cardiac death (SCD) and progressive left ventricular (LV) impairment are complications associated with hypertrophic cardiomyopathy (HCM).^1^ Interstitial fibrosis and scarring are linked with progressive LV dysfunction and are associated with SCD. Also abnormalities of myocardial perfusion are of importance in the pathophysiology of the disease. A few studies have used cardiovascular MRI (CMR) to describe LV perfusion abnormalities in patients with HCM, during adenosine stress^2,3^ or at rest.^4–6^ Abnormal stress perfusion has been related to the presence of microvascular disease. Conversely, abnormal rest perfusion has been connected to the degree of hypertrophy and the extent of fibrosis detected with late gadolinium enhancement (LGE).

The aims of the present study were to measure the prevalence of abnormal rest perfusion in a population of patients with HCM referred for CMR, and to define the relationship between rest perfusion abnormalities and the extent and pattern of LGE and the presence of risk factors for sudden death.

## Materials and methods

This multicentre study involved 80 consecutive patients with HCM referred for CMR from dedicated HCM clinics at the Ospedale di Rivoli (Rivoli, Torino, Italy; *n* = 56) and at the Ospedale Cardinal Massaia (Asti, Italy; *n* = 24) on the date of their yearly routine clinical follow-up. Patients with contraindications to CMR or history of anaphylactic reactions and patients with atrial fibrillation were excluded.

### Risk stratification

Four risk factors were used to stratify patients' risk for SCD: family history of HCM-related premature SCD, unexplained syncope, presence of severe LV hypertrophy (≥30 mm) and episodes of non-sustained ventricular tachycardia (NSVT) evaluated on an ECG-Holter performed on the enrolment visit (1 month before the CMR examination). Echocardiographic parameters were considered from the most recent available examination.

### Image acquisition

CMR was performed according to standard protocols^7,8^ using a 1.5 T system (Siemens Avanto, Erlangen, Germany) with fast gradients (45 mT/m; 200 T/m/s slew-rate), using a 12-element cardiac phased-array coil and cardiac package (Siemens Syngo-VB13, Erlangen, Germany). Steady-state free precession loops [repetition time (TR) 3.6 ms, echo time (TE) 1.8 ms, flip angle 65°, 30 acquired cardiac phases, typical voxel size after adapting the field of view to the anatomy of the chest of the patient 1.7 × 1.4 × 8 mm] were acquired in LV long-axis and short-axis views for LV mass and function measurements.

First-pass perfusion imaging was performed during injection of a gadolinium contrast agent (gadobutrol; Gadovist, Schering AG, Berlin, Germany) at a dose of 0.1 mmol/kg of body weight of gadolinium administered with a power injector (Medrad Spectris Solaris, Medrad Europe BV, Maastricht, The Netherlands) at a rate of 5 ml/s, followed by 20 ml saline. Perfusion images were acquired using a saturation-recovery sequence (prepulse delay 100 ms; turbo fast low-angle shot) with an acquisition time of 172 ms per section, TR 1.5 ms, TE 0.99 ms, flip angle 12°, typical voxel size after adapting the field of view 1.7 × 2.6 × 10 mm. Perfusion series were acquired in three short-axis views covering the basal, mid-ventricular and apical segments and additionally in a four-chamber long-axis view when heart rate allowed it. The minimum duration of acquisition of the perfusion sequence was 1 min, corresponding to 45–90 dynamics depending on rest heart rate. Free breathing was permitted after first passage of the contrast agent and the remaining dose was administered after the completion of the perfusion sequence (up to a total of 0.2 mmol/kg of body weight). LGE was performed approximately 20 min after the second injection using an inversion-recovery spoiled gradient-echo sequence (TR 2.4 ms, TE 0.96 ms, flip angle 15°, typical voxel size after adapting the field of view 1.8 × 1.3 × 8 mm).

### Image analysis

All images were analysed using a workstation (Argus; Siemens, Germany) by three of the authors whose joint opinion was reached in consensus. Segmentation into 16 myocardial segments^9^ was used to describe the findings. Wall thickness was measured in end-diastole at the point of maximum thickness of each segment. Basal and mid-ventricular segments were measured on short-axis views, apical segments were measured in four- and two-chamber long axis views, as appropriate. The endocardial and epicardial border of the LV were traced in each short-axis view in the end-diastolic and end-systolic frames, to measure LV global systolic function and LV mass. The area of the atria was measured in end-systole in the four-chamber views. Perfusion images were evaluated qualitatively by visual inspection. Abnormal rest perfusion was defined as a reduced wash-in of the contrast agent in relation to other parts of the myocardium, persisting for at least five heartbeats.^2^ The distribution of the perfusion defects was compared with the LV myocardial thickness and the distribution of LGE. LGE was evaluated visually (presence/absence; number of LGE positive segments). The pattern of LGE was scored according to Moon et al.^10^ Moreover, semi-automated quantification of LGE was performed according to published methods.^11–13^ LGE volume was computed based on a 5 SD threshold above the mean remote myocardial signal^13^ using dedicated software (CVI 42 version 4, Circle Cardiovascular Imaging, Calgary, Alberta, Canada).

### Statistical analysis and ethics

Patients with abnormal rest perfusion were compared with patients with normal rest perfusion for the presence of risk factors, echocardiographic parameters, and CMR findings. Continuous data are presented as mean ± SD; dichotomous data are presented as numbers and percentages. The Kolmogorov–Smirnov test was used for distribution checks of continuous variables. Differences in baseline characteristics between groups were evaluated with unpaired Student's *t*-tests, Mann–Whitney U-tests or Fisher's exact tests as appropriate. All tests were two sided, and a value of *p* < 0.05 was considered statistically significant. The association between perfusion abnormalities and hypertrophy was assessed with logistic regression. The association between prevalence of abnormal rest perfusion and LGE, hypertrophy, and the association between LGE and hypertrophy were performed with mixed effects models. All statistical analyses were carried out using the statistical software packages SAS 9.2 (SAS Institute, NC, USA) and SPSS v.19 (SPSS, Chicago, IL, USA). This study complies with the Declaration of Helsinki and was conducted according to the standards set by the institutional ethics committee. No changes to the clinical protocol in use for the evaluation of patients with HCM were needed to carry out the study; therefore, no formal approval from the institutional ethics committee was sought. Informed consent was obtained from all patients.

## Results

**Figure 1:**
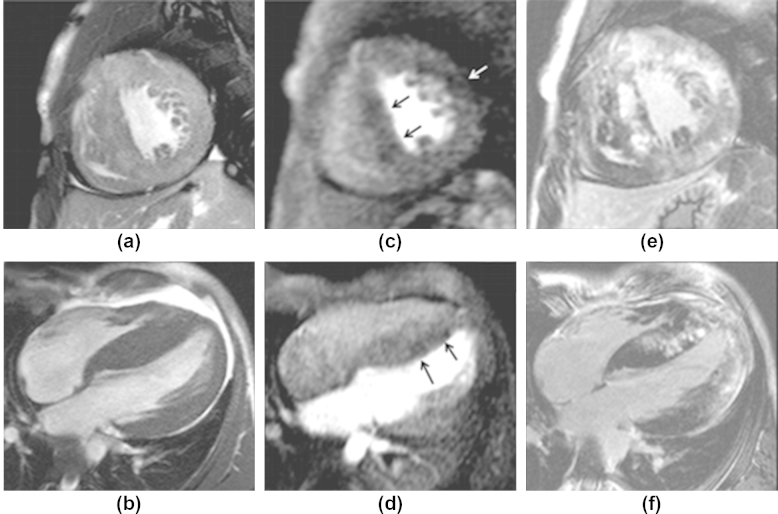
Findings in a 35-year-old patient with severe hypertrophy. (a) Short-axis and (b) long-axis images showing the segments with maximum hypertrophy (the short axis cine sequence shown (a) was acquired soon after gadolinium injection, showing raised signal in correspondence with the areas of enhancement seen in (e). Perfusion images at peak enhancement of normal myocardium, showing rest perfusion abnormalities in short-axis (c) and long-axis (d), corresponding to areas of LGE (e–f).

Abnormal rest perfusion was present in 24 patients (30%), involving 62 segments (2.6 segments per-patient among positives; range 1–10). Patients with normal and abnormal rest perfusion were identical for most demographics and echocardiographic characteristics after indexing for body surface area (BSA; Table 1). Patients with abnormal perfusion showed a higher indexed end-systolic volume, although the difference of LV ejection fraction was not significant (Table 2). All areas with abnormal rest perfusion showed LGE (Fig 1) with mid-myocardial localization. None of the patients showed any perfusion defects or LGE with patterns and distribution compatible with coronary artery disease.

### Comparison between perfusion and LGE

Sixty-four patients (80%) showed areas of LGE, with a total of 270 positive segments (21.1%; 3.4 ± 2.8 segments/patient; range 0–12). LGE was present in all patients with rest perfusion abnormalities and in 71% of those with normal rest perfusion (*p* = 0.003; Table 2). The prevalence of LGE was associated with the distribution of hypertrophy (*p* < 0.001) and with the prevalence of perfusion abnormalities (*p* < 0.001) in mixed-effects models.

Patients with abnormal rest perfusion presented with a more extensive LGE in comparison with patients with normal rest perfusion on both visual assessment (5.4 ± 2.9 LGE positive segments, range 1–12 segments versus 2.6 ± 2.3 segments, range 0–9 segments, respectively; *p* = 0.001; Table 2) and quantitative assessment (28% ± 17% versus 14%±12%, respectively; *p* = 0.001; Table 2). Finally, patients with abnormal rest perfusion showed a significantly higher prevalence of a confluent pattern of LGE (75% versus 38%; *p* = 0.002; Table 2).

### Comparison between perfusion and hypertrophy

The LV maximum thickness was 23 ± 7 mm (15–46 mm; patients with abnormal rest perfusion 16–40 mm; patients with normal rest perfusion 15–46 mm; *p* = 0.19), with a total of 442 hypertrophic segments of 1280 analysed (34.5%; 5.7 ± 3 per patient). The basal antero-septal segment showed the highest average maximal thickness (20.1 ± 7 mm), followed by the mid-ventricular infero-septal segment (18.5 ± 7 mm) and the anterior septum at mid-ventricular level (17.3 ± 8 mm). The localization of perfusion abnormalities was associated with the degree of hypertrophy in a mixed-effects model (*p* < 0.001).

### Prevalence of risk factors for SCD

Seventeen patients (21%) had two or more risk factors for SCD, 23 patients (29%) had one risk factor, and 40 patients (50%) had no risk factors. The average number of risk factors per patient in the groups with abnormal and normal rest perfusion was not different from the average of the population (Table 1). An association was found between the presence of rest perfusion abnormalities and the prevalence of NSVT (*p* = 0.03). No association was found between the presence of rest perfusion abnormalities and any of the other considered risk factors (Table 1).

A higher prevalence of NSVT was also associated with the presence of significant hypertrophy (50% amongst patients with maximum LV thickness >30 mm and 16% amongst the others; *p* = 0.002) and with the volume of LGE both for 2 SD and 5 SD (*p* = 0.03 and *p* = 0.022, respectively).

**Figure 2:**
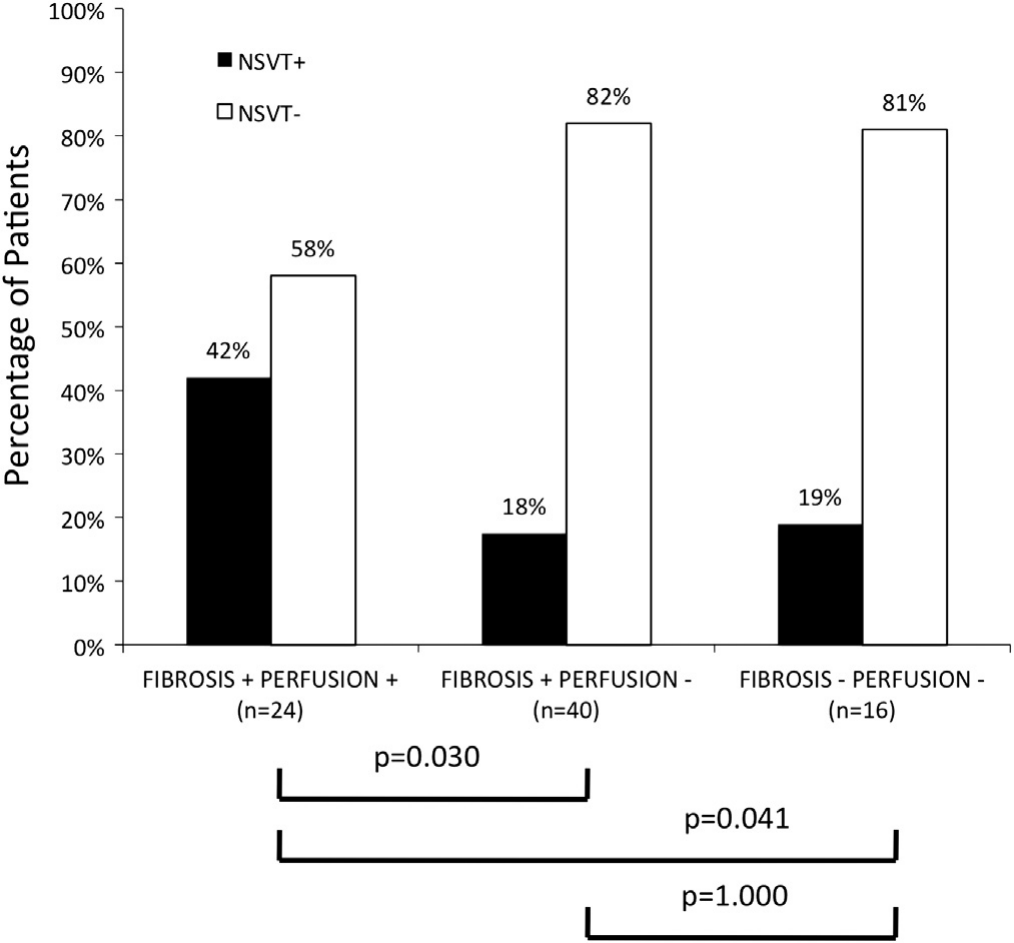
Percentage of patients with NSVT for three different groups based on the presence of absence of fibrosis (fibrosis+/fibrosis–) and rest perfusion abnormalities (perfusion+/perfusion–).

Fig 2 shows the percentage of patients with NSVT for three different patient categories based on the presence of scar tissue (fibrosis+/−) in combination with abnormal rest perfusion (perfusion+/−). Patients with fibrosis and rest perfusion abnormalities (fibrosis+/perfusion+) showed a prevalence of NSVT that was more than twice as high as in patients showing fibrosis with normal rest perfusion (fibrosis+/perfusion–; *p* = 0.03) and in patients without fibrosis and without rest perfusion abnormalities (fibrosis−/perfusion−; *p* = 0.04). Interestingly, no difference was found between patients with fibrosis and normal rest perfusion and patients without fibrosis and without rest perfusion abnormalities (*p* = 1.000).

## Discussion

The main findings of the present study are that (1) rest perfusion abnormalities are observed in 30% of patients with HCM; (2) rest perfusion abnormalities correlate with the severity of LGE and with the degree of hypertrophy; and (3) abnormal rest perfusion identifies a subgroup of patients with more advanced and clinically significant fibrosis.

The high prevalence of LGE in patients with HCM has been reported in many studies.^11,12,14^ LGE pattern and distribution also contribute to the differential diagnosis of HCM from other cardiomyopathies.^15,16^ LGE is associated with the degree of hypertrophy^17,18^ and has been associated with the occurrence of ventricular arrhythmias,^19,20^ progressive ventricular dilatation, markers of SCD,^10^ with all-cause and cardiac mortality,^11^ as well as with major cardiovascular events, hospitalization, heart failure, and arrhythmic events.^12^

In recent years, several authors have proposed the use of either rest or stress perfusion CMR for the assessment of patients with HCM. Matsunaka et al.^4^ was the first to describe a very high prevalence (75%) of rest perfusion abnormalities in a small selected population of 12 patients with HCM and related its presence to the distribution of LGE and the reduction of regional contractile function. The authors hypothesized a relationship between abnormal rest perfusion and the mechanical and anatomical effects of the increase in extracellular water fraction and the reduction in capillary density, respectively, observed in areas of LGE. In a different study, Soler et al.^6^ related abnormal rest perfusion (seen with a prevalence of 30%) to an impairment of systolic regional contraction. More recently, Melacini et al.^5^ demonstrated LGE and rest perfusion abnormalities in 46% of patients with HCM.

In the present study, abnormal rest perfusion was found in 30% of patients, in accordance with previous studies. To the authors' knowledge, the present study enrolled the largest population of patients so far. All perfusion abnormalities in the present cohort were spatially associated with regions of LGE. Perfusion defects were mostly associated with areas of confluent LGE. This likely represents an extreme degree of microvascular remodelling in areas of more dense scar,^21,22^ resulting in a slower inflow of contrast agent during first-pass.^23^ Accordingly, more severe fibrosis relates to more pronounced microvascular remodelling and to a higher probability of rest perfusion abnormalities. Quantitative LGE analysis for 5 SD threshold confirmed the significant correlation between rest perfusion abnormalities and volume of LGE. Therefore, based on the present results, rest perfusion abnormalities should not be considered an expression of myocardial ischaemia but rather a marker of severe myocardial fibrosis.

Although the present study focused on rest perfusion abnormalities, adenosine-induced perfusion abnormalities have also been reported in the literature.^2,3^ These are considered instead a marker of microvascular dysfunction and have also been associated in previous studies with the degree of hypertrophy. However, adenosine-induced perfusion abnormalities have a subendocardial localization that does not resemble the distribution of LGE,^2^ probably indicating a different underlying pathophysiological mechanism.

The possibility that severe myocardial fibrosis can cause perfusion abnormalities should, however, be considered when adenosine stress perfusion imaging is prescribed in patients with HCM to assess the LV ischaemic burden. Scar-related perfusion abnormalities and adenosine-induced perfusion abnormalities are, in fact, likely to coexist in a significant proportion of patients. However, the true ischaemic burden is likely to be better represented by adenosine-induced perfusion defects, without considering areas of abnormal perfusion correlated with severe myocardial fibrosis. Combined high-resolution quantification of stress perfusion and LV fibrosis could in future improve the assessment of patients with HCM and perhaps even enable the use of perfusion as a novel independent predictor of events.

Currently, risk stratification schemes identify only a limited proportion of patients at risk of SCD,^1,24–28^ which is the most threatening expression of the disease. LGE has been proposed as a method to improve risk stratification and CMR is being used in an increasing number of patients.^29,30^ However, the reported high prevalence of LGE among patients with HCM and the relatively low incidence of arrhythmic events at follow-up^31^ clearly delineates an important overlap between groups of patients with different degrees of myocardial damage and risk,^32^ underscoring the need to improve risk stratification beyond the mere presence or absence of LGE.^33^

The correlation between imaging findings and risk factors for SCD was examined in the present cohort population. Patients with abnormal rest perfusion and LGE had more than double the prevalence of NSVT when compared with the group of patients showing LGE and normal rest perfusion. The latter did not differ from the group of patients without LGE (Fig 2). As such, abnormalities of rest perfusion could be a useful marker to identify those patients with more severe microvascular remodelling, and severe and dense scarring. As rest perfusion examination is an easily applicable, rapid, and reproducible technique, it is feasible to obtain important information from its regular application at the expense of a trivial time delay.

### Study limitations

The present findings are based on a population of patients with a clinical profile of low–intermediate risk. The validity of these findings in a population of high-risk patients with HCM should be further evaluated. Quantitative analysis of rest perfusion abnormalities was not performed but visual assessment was performed; the perfusion acquisition protocol was optimized for this purpose. A stress perfusion study was also not performed.

In conclusion, first-pass perfusion CMR can identify abnormal rest perfusion in a significant proportion of patients with HCM. These abnormalities are associated with areas of severe fibrosis and are most likely due to a reduction in the number of capillaries in areas of scar tissue. Adequately powered prospective studies relating the CMR findings, and in particular, the presence of rest perfusion abnormalities, to risk stratification would be needed in future.

## Figures and Tables

**Table 1:** Characteristics, demographics, and risk factors for sudden cardiac death of the study group and comparison between patients with abnormal and normal rest perfusion.

	All patients (*n* = 80)	Group 1 (abnormal rest perfusion, *n* = 24)	Group 2 (normal rest perfusion, *n* = 56)	*p*-Value
Demographics				
Age	49.8 ± 18	45.9 ± 16	51.3 ± 18	0.250
Male sex	53 (70%)	18 (82%)	35 (65%)	0.176
Height, cm	169.4 ± 10	174.1 ± 10	167.5 ± 9	0.010
Weight, kg	73.1 ± 15	77.7 ± 11	71.1 ± 16	0.082
Body mass index, kg/m^2^	25.6 ± 4	25.6 ± 3	25.6 ± 5	0.926
Body surface area, m^2^	1.9 ± 0	1.9 ± 0	1.8 ± 0	0.018
New York Heart Association functional class	1.4 ± 1	1.4 ± 1	1.3 ± 1	0.834
Echocardiographic parameters				
Mitral regurgitation	36 (47%)	11 (50%)	25 (46%)	0.443
LV outflow tract obstruction	27 (35%)	11 (50%)	16 (30%)	0.102
Systolic anterior movement mitral valve	25 (33%)	9 (40%)	16 (30%)	0.601
Echocardiographic ejection fraction	57.3 ± 19	56.3 ± 20	57.7 ± 18	0.782
Risk factors for sudden cardiac death				
Family history of HCM-related sudden cardiac death, %	22.4	13.6	25.9	0.364
Syncope, %	11.8	9.1	13	1.000
Non-sustained ventricular tachycardia (Holter monitoring), %	22.4	40.9	14.8	0.030
Severe hypertrophy (>30 mm)	17.1%	22.7%	14.8%	0.504
Number of risk factors for sudden cardiac death	0.8 ± 1	0.9 ± 1	0.7 ± 1	0.470

LV, left ventricular, HCM, hypertrophic cardiomyopathy.

**Table 2:** Cardiovascular MRI (CMR) measurements of the study group and comparison between patients with abnormal and normal rest perfusion.

	All patients (*n* = 80)	Group 1 (abnormal rest perfusion, *n* = 24)	Group 2 (normal rest perfusion, *n* = 56)	*p*-Value
Left ventricular function and mass				
Indexed left atrium (cm^2^/m^2^)	16 ± 4	17 ± 5	15 ± 4	0.107
Indexed right atrium (cm^2^/m^2^)	12 ± 3	12 ± 2	11 ± 3	0.218
Ejection fraction: left ventricle	58.6 ± 9	55.4 ± 10	59.9 ± 9	0.069
Indexed stroke volume (ml/beat/m^2^)	45.4 ± 8	45.4 ± 8	45.3 ± 8	0.920
Indexed end-diastolic volume: left ventricle (ml/m^2^)	79.1 ± 15	84.6 ± 16	77.0 ± 15	0.070
Indexed end-systolic volume: left ventricle (ml/m^2^)	34.0 ± 13	39.2 ± 14	32.0 ± 12	0.038
Indexed left ventricular wall mass (g/m^2^)	102.3 ± 40	116.5 ± 46	96.6 ± 36	0.066
Maximum left ventricular myocardial thickness	23.0 ± 7	24.6 ± 7	22.4 ± 7	0.194
Late gadolinium enhancement				
Presence of fibrosis	64 (80%)	24 (100%)	40 (71%)	0.003
Number of segments with fibrosis	3.4 ± 3	5.4 ± 3	2.6 ± 2	0.001
Fibrosis pattern				0.007
Confluent	39 (49%)	18 (75%)	21 (38%)	0.002
Diffuse	14 (18%)	3 (13%)	11 (14%)	0.441
Mixed	11 (14%)	3 (13%)	8 (14%)	0.832
Fibrosis % volume (5 SD above mean of normal myocardium)	18 ± 15	28 ± 17	14 ± 12	0.001
